# Biochemical Evidence of Acute Hormonal Abnormality in Aneurysmal Subarachnoid Hemorrhage: Correlation with Clinical Severity

**DOI:** 10.3390/ijms27093732

**Published:** 2026-04-22

**Authors:** Ghaith Saleh R. Aljboor, Aoun Tulemat, Hilali Ahmed, Mugurel Petrinel Rădoi, Toader Corneliu, Toma Marius Papacocea

**Affiliations:** 1 Department of Neurosurgery, National Institute of Neurology and Neurovascular Diseases, 020021 Bucharest, Romania; 2 Department of Neurosurgery, University of Medicine and Pharmacy “Carol Davila”, 020021 Bucharest, Romania

**Keywords:** aneurysmal subarachnoid hemorrhage, acute hormonal abnormalities, pituitary-adrenal axis, acth-axis biochemical abnormality, neuroendocrine abnormality, glasgow coma scale, hunt and hess scale, fisher grade, critical care neurosurgery, prognostic biomarkers

## Abstract

Aneurysmal subarachnoid hemorrhage (aSAH) is a life-threatening condition with high morbidity among survivors. Emerging evidence suggests that acute biochemical hypothalamic–pituitary axis disturbances, resulting from disruption of neuroendocrine regulation, are an underrecognized complication in the acute phase of aSAH. However, its correlation with clinical severity remains insufficiently explored. To investigate whether clinical severity of aSAH predicts acute biochemical pituitary-axis abnormalities and identify which hormonal axes are most affected in the acute phase. A prospective observational study was conducted at The National Institute of Neurology and Neurovascular Diseases, Bucharest (October 2024–March 2025) on 38 patients confirmed aSAH admitted within 48 h of symptom onset, of which 20 patients were included. Hormonal panels assessing adrenocorticotropic hormone (ACTH), growth hormone (GH), thyroid-stimulating hormone (TSH), and antidiuretic hormone (ADH) were obtained prior to surgical intervention. Clinical severity was evaluated using the Glasgow Coma Scale (GCS), the Hunt and Hess (HH) scale, and the Modified Fisher Scale. Correlations between hormonal deficiencies and severity scores were analyzed using the Spearman correlation. Biochemical abnormality of the ACTH axis was most prevalent (75%), followed by ADH (50%) and TSH (40%), while GH deficiency was rare (5%). ACTH-axis biochemical abnormality correlated significantly with lower GCS (ρ = −0.61, *p* = 0.004) and higher HH scores (ρ = 0.59, *p* = 0.006). Multiple-axis abnormalities demonstrated the strongest correlations with all severity metrics (GCS: ρ = −0.68, *p* = 0.001; HH: ρ = 0.72, *p* < 0.001; Fisher: ρ = 0.57, *p* = 0.009). Greater clinical severity in aSAH is associated with a higher prevalence of acute biochemical endocrine abnormalities, particularly involving the ACTH axis and multiple hormonal pathways. These findings are exploratory and hypothesis-generating. Early hormonal assessment in patients with severe aSAH may help identify individuals at risk for acute endocrine abnormality, but validation in larger prospective studies is required before influencing clinical practice.

## 1. Introduction

### 1.1. Background

Aneurysmal subarachnoid hemorrhage (aSAH) is a life-threatening cerebrovascular event caused by rupture of an intracranial aneurysm, resulting in bleeding into the subarachnoid space. The global incidence of aSAH is estimated at 6.1 per 100,000 person-years, with approximately 8.09 million prevalent cases worldwide [1]. aSAH primarily affects middle-aged to older adults and is strongly associated with modifiable risk factors, including hypertension, smoking, alcohol consumption, and family history [2].

Despite advances in neurosurgical and endovascular management, aSAH remains associated with high morbidity and mortality. Approximately one-third of survivors experience lifelong systemic and neurological complications [3]. Recent epidemiological studies highlight the significant healthcare burden of SAH, yet endocrine complications such as hypopituitarism remain underrecognized in both clinical practice and research [4].

Acute pituitary hormonal abnormality is a clinically significant but often overlooked consequence of aSAH [4]. Ischemic and mechanical insults disrupt the hypothalamic-pituitary axis, ultimately leading to acute hormonal disturbances that may reflect transient biochemical abnormalities during critical illness [5].

### 1.2. Pathophysiology of Pituitary Abnormality in SAH

Pituitary abnormality affects 37.5–55% of aSAH survivors, most commonly impacting growth hormone, gonadotropins, and adrenal function [6,7]. Multiple mechanisms contribute to acute pituitary hormonal axis abnormality by directly or indirectly damaging the pituitary gland and hypothalamus. The hypophyseal portal system is highly susceptible to ischemic injury due to global cerebral hypoperfusion, elevated intracranial pressure, and vasospasm following SAH [8]. Reduced oxygenation impairs pituitary hormone synthesis and secretion. Additionally, the pituitary’s anatomical proximity to the circle of Willis makes it vulnerable to compression from subarachnoid blood accumulation [9]. Direct hemorrhagic insult and raised intracranial pressure further compromise the hypothalamic-pituitary axis.

SAH also triggers systemic and localized inflammation. Elevated pro-inflammatory cytokines, including tumor necrosis factor-alpha (TNF-α) and interleukin-6 (IL-6), disrupt hormone secretion and promote apoptosis of pituitary cells. Excessive apoptosis of hormone-secreting cells results in deficiencies and impaired stress responses, often irreversible due to the pituitary’s limited regenerative capacity [10].

A clinically relevant consequence may be transient dysregulation of the hypothalamic–pituitary–adrenal axis, which can manifest as low ACTH and cortisol values, causing hyponatremia, hypotension, and increased vulnerability to critical illness [5]. These metabolic derangements can worsen neurological outcomes and complicate acute management.

### 1.3. Rationale and Knowledge Gap

Recognition of post-SAH pituitary abnormality is essential given its implications for patient recovery, functional independence, and long-term quality of life. Hypopituitarism leads to numerous complications in SAH survivors, including chronic fatigue, increased cardiovascular risk, cognitive impairment, and metabolic abnormalities [11]. Despite evidence suggesting that early hormonal abnormality correlates with prolonged hospital stays and poorer outcomes, pituitary function is not routinely assessed in the acute phase of SAH [12].

Understanding the relationship between aSAH severity and acute hypopituitarism is crucial for early identification and intervention. If direct correlations exist, targeted screening protocols could be developed to improve patient outcomes, prevent adverse events, and optimize long-term prognosis.

Several critical knowledge gaps remain. First, the prevalence and severity of acute hypopituitarism post-SAH are not well established. Second, there are no validated predictive models to determine which patients are at higher risk for developing acute hypopituitarism. Third, the impact of early hormonal deficiencies on neurological recovery and outcomes has not been fully investigated. Finally, there are no formal screening guidelines or management protocols for post-SAH endocrine abnormalities.

By addressing these knowledge gaps, this study aims to provide insights into the interplay between aSAH severity and acute pituitary hormonal abnormality, potentially informing future clinical protocols.

### 1.4. Study Objectives

This study aims to explore the association between aSAH severity and the presence of acute pituitary hormonal abnormality. Specific objectives include the following:

1. Establish the prevalence of acute pituitary abnormality in aSAH patients during the acute phase.
2. Evaluate the relationship between aSAH severity indicators (Glasgow Coma Scale, Hunt and Hess scale, Modified Fisher grade) and acute biochemical pituitary axis abnormality.
3. Identify which hormonal axes are most affected in acute aSAH.
4. Determine the clinical utility of routine endocrine screening in acute aSAH management.

## 2. Results

### 2.1. Patient Characteristics

The cohort included 20 patients with aSAH (14 females, 6 males; F:M ratio 2.3:1). The median age was 63.5 years (IQR: 54–68 years; mean: 59.2 ± 10.3 years). Regarding comorbidities, 14 patients (70%) had a history of hypertension, 6 patients (30%) had diabetes mellitus, and 3 patients (15%) had a history of epilepsy. Notably, 17 patients (85%) were active smokers at presentation. Body mass index data showed that 60% of patients were either overweight (BMI 25–29.9 kg/m^2^) or obese (BMI ≥ 30 kg/m^2^).

Overall, the demographic profile highlights a predominantly middle-aged population with a high burden of vascular risk factors, particularly hypertension and smoking, which are important contributors to aSAH risk.

### 2.2. Hormonal Abnormality Findings

Biochemical hormonal profile evaluation in the acute phase revealed a high prevalence of endocrine abnormalities across multiple pituitary axes (Table 1). Given the small sample size and multiple correlation analyses performed, these associations should be interpreted as exploratory.

Adrenocorticotropic hormone (ACTH): abnormal ACTH values were the most prevalent, observed in 15 patients (75%). The mean ACTH level was 13.9 ± 18.7 pg/mL. Although the mean value was above the lower reference limit, a significant subset had ACTH values below the laboratory reference range, indicating acute biochemical HPA-axis disturbance during severe illness rather than definitive adrenal insufficiency.

Thyroid-stimulating hormone (TSH): TSH abnormalities were present in 8 patients (40%), with a mean TSH level of 0.91 ± 0.62 μIU/mL, which is toward the lower end of the normal range.

Antidiuretic hormone (ADH): Low ADH activity, reflected by reduced FIA levels, was identified in 10 patients (50%). The mean FIA concentration was 68.2 ± 42.1 pg/mL, indicating a moderate reduction relative to expected stress-induced levels. The mean FPD was 295.6 ± 8.3 mOsmol/kg, and no patients exhibited abnormally low FPD values.

Growth hormone (GH): abnormal GH values were the least common, affecting only 1 patient (5%). The mean GH level was 0.79 ± 0.41 ng/mL, within low-normal limits.

In this cohort, hormonal abnormalities were predominantly seen in the adrenal (ACTH), vasopressin (ADH), and thyroid (TSH) axes, with growth hormone disturbances being less frequent. However, no patient met clinical or biochemical criteria for diabetes insipidus or SIADH.

### 2.3. Correlations Between Hormonal Abnormalities and Clinical Severity

**Figure 1:**
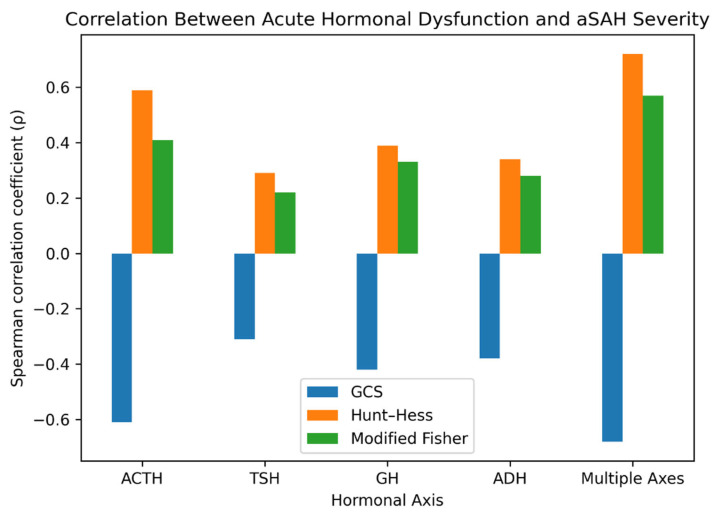
Spearman correlation coefficients (ρ) between biochemical hormonal-axis abnormalities and clinical severity scores (n = 20). Significant correlations included ACTH with GCS (ρ = −0.61, *p* = 0.004) and Hunt and Hess (ρ = 0.59, *p* = 0.006), and multiple-axis abnormalities with GCS (ρ = −0.68, *p* = 0.001), Hunt and Hess (ρ = 0.72, *p* < 0.001), and Modified Fisher (ρ = 0.57, *p* = 0.009). Analysis was based on complete-case data, with hormonal abnormalities treated as binary variables using institutional reference ranges. No outlier exclusion or multiple comparison correction was applied.

**Figure 2:**
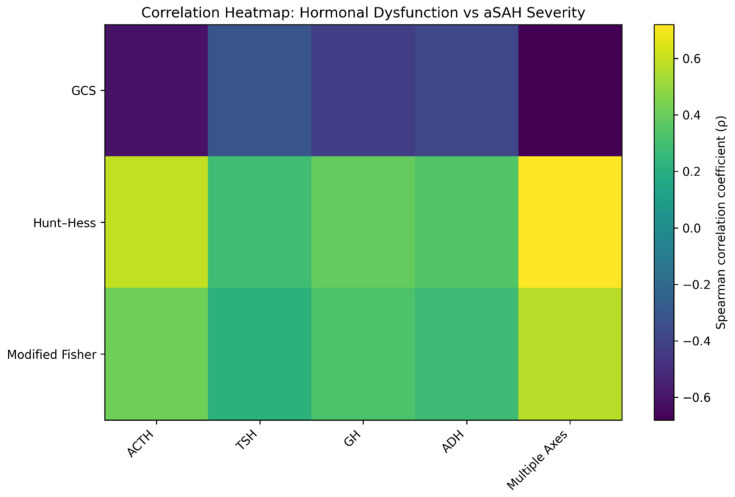
Heatmap of Spearman correlation coefficients (ρ) demonstrating associations between hormonal-axis abnormalities and clinical severity scores (n = 20). Strongest correlations were observed for multiple-axis abnormalities with Hunt and Hess (ρ = 0.72) and GCS (ρ = −0.68), and for ACTH with GCS (ρ = −0.61). All variables were analyzed without transformation or outlier removal; results are presented descriptively.

**Figure 3:**
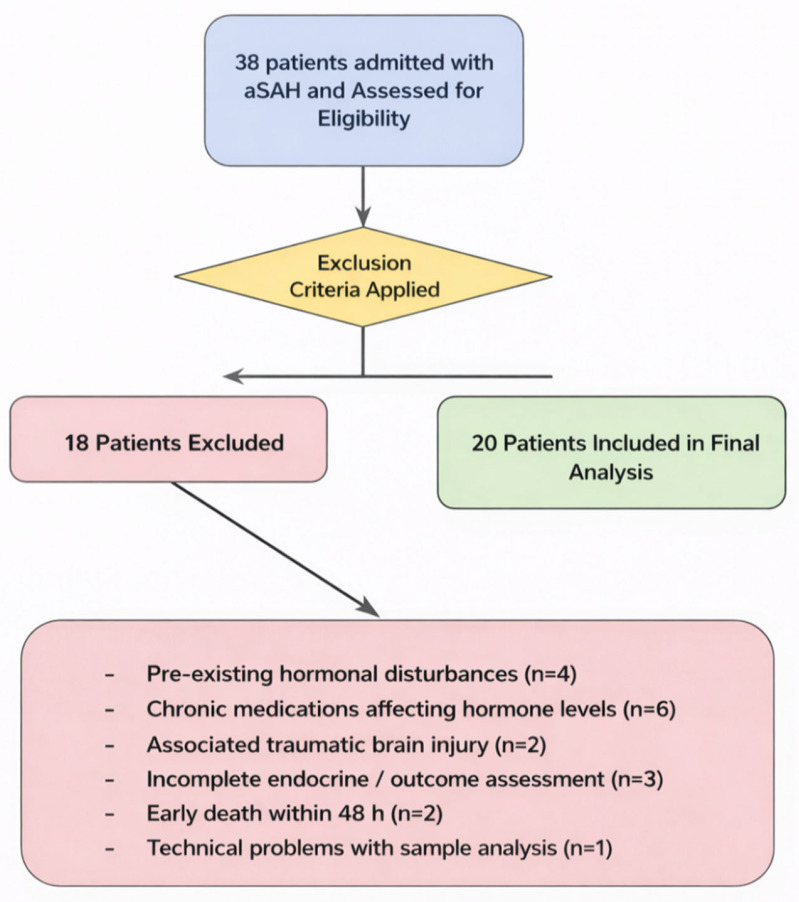
A CONSORT-style flowchart shows the patient’s selection process. Of 38 patients with confirmed aSAH screened between October 2024 and March 2025, 20 met the inclusion criteria and completed hormonal assessment. Exclusions (n = 18) were based on predefined criteria, including incomplete hormonal screening, pre-existing endocrine disorder, or death within 48 h (since admission). All included patients (n = 20) had complete datasets for clinical severity scores and baseline hormonal assessment; no imputation was performed.

Given the limited sample size and the number of exploratory correlation analyses performed, these associations should be interpreted cautiously as hypothesis-generating signals rather than definitive inferential findings. Correlation analyses revealed significant associations between hormonal abnormalities and clinical severity metrics (Table 2, Figure 1, Figure 2 and Figure 3).

#### 2.3.1. GCS and Hormonal Abnormalities

ACTH-axis biochemical abnormality was significantly associated with lower GCS scores (ρ = −0.61, *p* = 0.004, 95% CI: −0.83 to −0.24), indicating that patients with more severe neurological impairment were more likely to have ACTH suppression. GH abnormality showed a moderate inverse correlation with GCS (ρ = −0.42, *p* = 0.048, 95% CI: −0.70 to −0.01). Multiple-axis hormone abnormalities demonstrated the strongest relationship with GCS scores (ρ = −0.68, *p* = 0.001, 95% CI: −0.86 to −0.36), suggesting that patients with the most severe neurological presentations had abnormalities in multiple hormonal axes (Table 3). ADH abnormality showed a trend toward association with lower GCS (ρ = −0.38, *p* = 0.073, 95% CI: –0.68 to 0.04), while TSH abnormality exhibited no significant correlation (ρ = −0.31, *p* = 0.12, 95% CI: −0.64 to 0.10).

#### 2.3.2. Hunt and Hess Scale and Hormonal Abnormalities

ACTH-axis biochemical abnormality correlated significantly with higher Hunt and Hess scores (ρ = 0.59, *p* = 0.006, 95% CI: 0.20 to 0.82), indicating that more severe clinical grades were associated with ACTH-axis biochemical abnormality. GH abnormality also correlated with elevated HH scores (ρ = 0.39, *p* = 0.033, 95% CI: 0.03 to 0.67). Multiple-axis abnormalities showed the strongest association with HH scores (ρ = 0.72, *p* < 0.001, 95% CI: 0.45 to 0.87). ADH abnormality was modestly associated with higher HH scores (ρ = 0.34, *p* = 0.045, 95% CI: −0.02 to 0.63), while TSH abnormality showed no significant relationship (ρ = 0.29, *p* = 0.23, 95% CI: –0.09 to 0.60).

#### 2.3.3. Modified Fisher Scale and Hormonal Abnormalities

Biochemical abnormality of the ACTH axis showed a trend toward correlation with higher Fisher grades (ρ = 0.41, *p* = 0.065, 95% CI: 0.03 to 0.69). GH abnormality demonstrated a near-significant trend with Fisher scale (ρ = 0.33, *p* = 0.058, 95% CI: −0.04 to 0.63). Multiple-axis abnormalities showed a significant association with Fisher grades (ρ = 0.57, *p* = 0.009, 95% CI: 0.18 to 0.80). ADH abnormality was not significantly correlated with Fisher grade (ρ = 0.28, *p* = 0.084, 95% CI: −0.10 to 0.59), and TSH abnormality showed no significant relationship (ρ = 0.22, *p* = 0.17, 95% CI: −0.16 to 0.55).

## 3. Discussion

### 3.1. Principal Findings

This prospective study demonstrates that greater clinical severity of aSAH is associated with biochemical abnormalities of the hypothalamic–pituitary axis in the acute phase, particularly involving the ACTH axis and multiple hormonal pathways. ACTH-axis biochemical abnormality was the most prevalent hormonal abnormality (75%), followed by ADH (50%) and TSH (40%), while GH abnormality was rare (5%). Importantly, ACTH-axis biochemical abnormality and multiple-axis abnormalities showed strong correlations with all three-severity metrics: GCS, Hunt and Hess scale, and Modified Fisher grade. Patients with multiple hormonal abnormalities exhibited the most severe clinical profiles, suggesting that cumulative endocrine abnormalities may serve as both a marker and mediator of poor outcomes. Those preliminary findings suggest a potential biochemical hormonal abnormality of the pituitary and, while not diagnostic, provide a basis for hypothesis generation and further investigation.

### 3.2. Comparison with Existing Literature

Our findings align with and extend previous research on post-SAH hypopituitarism. While chronic pituitary abnormality following SAH has been well documented, with prevalence rates of 37.5–55% in long-term survivors [6,7], our study focuses on the acute phase, which remains less well characterized. The high prevalence of ACTH-axis biochemical abnormality (75%) in our cohort exceeds rates reported in some chronic studies, suggesting that acute abnormalities may be more common than previously recognized or that some abnormalities are resolved over time.

The strong inverse correlation between ACTH levels and GCS scores (ρ = −0.61, *p* = 0.004) and positive association with Hunt and Hess scale (ρ = 0.59, *p* = 0.006) support the hypothesis that greater hemorrhage severity leads to more profound disruption of the hypothalamic-pituitary axis. These correlations are consistent with pathophysiological mechanisms, including ischemic injury to the hypophyseal portal system, mechanical compression from subarachnoid blood, and inflammatory-mediated pituitary cell apoptosis [8,9,10].

Previous studies have emphasized the delayed consequences of pituitary abnormality [5,12], but our analysis suggests that acute endocrine assessment may have immediate clinical utility. Early identification of ACTH-axis biochemical abnormality may justify closer endocrine surveillance and further confirmatory evaluation, although the therapeutic implications remain uncertain and require prospective validation [11].

### 3.3. Clinical Implications

The strong associations between severity metrics and hormonal abnormality have important clinical implications. First, patients presenting with severe aSAH (GCS ≤ 11, Hunt and Hess ≥ 3, or Modified Fisher ≥ 3) may be associated with a higher likelihood of acute neuroendocrine disturbance. Second, routine measurement of ACTH and cortisol in the acute phase may help identify patients at higher risk of biochemical abnormality of the ACTH axis; however, the clinical utility of early endocrine screening remains unproven and requires validation in larger studies.

Although these findings suggest that patients with severe aSAH are at increased risk of acute endocrine abnormality, they should be regarded as hypothesis-generating and insufficient to support changes to clinical guidelines without further validation.

### 3.4. Pathophysiological Considerations

The predominance of the acute biochemical abnormalities of the ACTH axis in our cohort likely reflects the hypothalamic–pituitary–adrenal axis’s particular vulnerability to ischemic and inflammatory insults. The corticotropes of the anterior pituitary have high metabolic demands and are highly sensitive to hypoperfusion [8]. Additionally, the proximity of the pituitary stalk to major cerebral vessels makes it susceptible to compression and shearing forces during acute SAH [9].

The correlation between hemorrhage severity (as measured by Fisher grade) and hormonal abnormality supports the hypothesis that greater blood burden in the subarachnoid space leads to more extensive pituitary compression and inflammatory activation [10]. The systemic inflammatory response, characterized by elevated cytokines such as TNF-α and IL-6, may directly suppress ACTH secretion and promote apoptosis of hormone-secreting cells.

The relatively low prevalence of GH abnormality in the acute phase (5%) contrasts with higher rates reported in chronic studies [6], suggesting that GH abnormality may develop gradually or that acute assessment using basal levels lacks sensitivity. This discrepancy highlights a limitation of our study and the broader challenge of diagnosing GH abnormality in critically ill patients.

### 3.5. Study Limitations

Several limitations warrant careful consideration. The small sample size (n = 20) limited statistical power and increased the risk of both type I and type II errors, particularly given the multiple exploratory correlation analyses performed without formal correction for multiple comparisons. Accordingly, the reported associations should be interpreted as hypothesis-generating signals rather than definitive inferential findings. In addition, the single-center design may limit external validity, as patient populations, aneurysm characteristics, and management protocols may differ across institutions.

The absence of dynamic hormonal testing represents a major methodological limitation. Basal hormone levels—particularly within the ACTH and GH axes—may not accurately distinguish transient critical illness–related endocrine responses from clinically meaningful pituitary insufficiency. Although stimulation tests such as insulin tolerance testing, ACTH stimulation, or CRH stimulation would provide more robust endocrine characterization, these were not feasible in the acute neurocritical care setting because of hemodynamic instability and impaired consciousness. Because basal ACTH and ADH measurements were obtained during severe acute neurological illness, some abnormalities may represent stress-related adaptive neuroendocrine responses rather than persistent hypothalamic-pituitary abnormality.

The wide dispersion of ACTH values, reflected by a standard deviation exceeding the mean, suggests substantial biological heterogeneity and further limits the stability of prevalence estimates, particularly for the high frequency of ACTH-axis abnormalities observed in this cohort. In addition, exclusion of patients who died within 48 h may have introduced survival bias by underrepresenting the most severe cases of aSAH, potentially affecting the observed correlations between endocrine abnormalities and severity metrics.

The lack of long-term follow-up prevents assessment of whether these early biochemical abnormalities persist, resolve, or predict chronic post-SAH endocrine sequelae. Longitudinal studies following hormonal trajectories from the acute phase through recovery would provide important insight into their clinical significance.

Finally, the absence of a control group limits causal inference. While the observed correlations between severity and hormonal abnormalities are biologically plausible, they do not establish causation. Future prospective controlled studies with matched comparator groups would strengthen causal interpretation and help distinguish disease-specific endocrine alterations from generalized critical illness responses.

### 3.6. Future Directions

Several research priorities emerge from this work. First, larger prospective multi-center studies are needed to validate our findings and establish evidence-based screening protocols. Second, investigation of whether early hormonal intervention (e.g., corticosteroid replacement in ACTH-abnormality patients) improves clinical outcomes would inform treatment guidelines. Third, longitudinal studies tracking hormonal function from the acute phase through long-term recovery would clarify the natural history and persistence of post-SAH hypopituitarism. Fourth, exploration of biomarkers that might predict hormonal abnormality (e.g., inflammatory cytokines, neuroimaging features) could refine risk stratification. Finally, cost-effectiveness analyses of routine endocrine screening in aSAH would support resource allocation decisions.

## 4. Methods

### 4.1. Study Design and Setting

This prospective observational study was conducted at The National Institute of Neurology and Neurovascular Diseases, Bucharest, from October 2024 to March 2025. The study protocol was approved by the institutional ethics committee, and written informed consent was obtained from patients or their legal representatives prior to enrollment.

### 4.2. Patient Selection

Inclusion criteria:

1. Age ≥ 18 years;
2. Confirmed aneurysmal subarachnoid hemorrhage haemorrhage on CT and angiography;
3. Admission within 48 h of symptom onset;
4. Availability of complete hormonal assessment prior to intervention.

Exclusion criteria:

1. Pre-existing pituitary or endocrine disorders;
2. Chronic corticosteroid or hormone therapy;
3. Non-aneurysmal SAH or associated traumatic brain injury;
4. Incomplete clinical or hormonal data;
5. Death within 48 h of admission.

Of 38 patients admitted with aSAH during the study period, 18 were excluded based on these criteria, leaving 20 patients for final analysis.

### 4.3. Clinical Assessments

#### 4.3.1. SAH Severity Classification

The severity of aSAH was assessed using three validated grading systems at admission. The Hunt and Hess Scale categorizes patients based on clinical severity, ranging from Grade 1 (asymptomatic or mild headache) to Grade 5 (deep coma, moribund). The Glasgow Coma Scale (GCS) provided an objective neurological assessment based on eye, verbal, and motor responses (range: 3–15). The Modified Fisher Scale evaluated hemorrhage haemorrhage extent on CT imaging (Grade 0–4), with higher grades predicting increased risk of delayed cerebral ischemia. These classifications were recorded at admission and analyzed for association with acute biochemical pituitary axis abnormality.

#### 4.3.2. Hormonal Assessment

Blood samples were obtained within 24 h of admission and prior to surgical intervention. Sampling was targeted between 08:00 and 10:00 to minimize circadian variation; however, minor deviations were unavoidable in the acute neurocritical care setting.

Basal serum levels of the following hormones were measured:

− Adrenocorticotropic hormone (ACTH);
− Growth hormone (GH);
− Thyroid-stimulating hormone (TSH);
− Antidiuretic hormone (ADH).

ADH was assessed using fluorescence immunoassay (FIA). Plasma osmolality, measured as functional plasma density (FPD), was used as a supportive physiological parameter rather than a direct measure of ADH concentration. FPD was not used to define hormonal abnormality but served as a physiological contextual marker of water-balance status.

Hormonal abnormalities were defined using the institutional laboratory reference ranges available for each assay platform. The thresholds used were as follows: ACTH 7.2–63.3 pg/mL, TSH 0.27–4.2 μIU/mL, GH 0.5–2.47 ng/mL, and ADH 0.87–30.58 pg/mL (converted from assay-provided SI units). Values below or above these ranges were categorized as biochemical abnormalities. Because critical illness may alter basal endocrine physiology, these thresholds were used as screening biochemical reference points rather than definitive diagnostic criteria.

Dynamic stimulation testing (e.g., ACTH stimulation or insulin tolerance testing) was not feasible due to patient instability and safety concerns. It is acknowledged that basal hormone measurements alone, —particularly for GH and the adrenal axis,- are insufficient for definitive diagnosis in critically ill patients and may result in misclassification of endocrine abnormality.

Patients were categorized as having no hormonal abnormality, single-axis abnormality, or multiple-axis abnormality (≥2 axes affected).

#### 4.3.3. Imaging Analysis

All participants underwent standardized neuroimaging protocols on admission, comprising non-contrast CT of the brain to confirm aSAH diagnosis, followed by either CT angiography or DSA for aneurysm localization and characterization. Aneurysm location, size, and hemorrhage haemorrhage distribution were documented by experienced neuroradiologists blinded to hormonal results.

### 4.4. Outcome Measures

The primary outcome was the presence of acute biochemical pituitary axis abnormality, defined as biochemical evidence of abnormality in one or more pituitary hormone axes during the acute phase (within 48 h) following aSAH. All hormone values were interpreted using reference ranges appropriate for critically ill patients. Secondary outcomes included the correlation between hormonal abnormalities and clinical severity scores.

### 4.5. Statistical Analysis

Statistical analysis was conducted using Python (version 3.11) with relevant libraries (pandas, scipy, statsmodels, and seaborn). Continuous variables were assessed for normality using the Shapiro–WilkShapiro-Wilk test. Normally distributed variables were presented as mean ± standard deviation (SD), while non-normally distributed data were described using median and interquartile range (IQR). Categorical variables were summarized as frequencies and percentages.

Comparisons between groups were performed using the independent samples t-test for normally distributed continuous variables and the Mann–WhitneyMann-Whitney U test for non-parametric data. Categorical variables were compared using Fisher’s exact test due to small sample sizes.

Correlations between hormonal levels and clinical severity scores (GCS, Hunt and Hess, Modified Fisher) were assessed using Spearman’s rank correlation coefficient (ρ) for non-parametric data. Each correlation analysis included reporting of the correlation coefficient, the corresponding two-tailed *p*-value, and 95% confidence interval (CI).

A two-sided *p*-value < 0.05 was considered statistically significant. Missing data were handled by listwise deletion; no data imputation was applied. All analysis wasanalyses were based only on available complete datasets for each variable. Given the number of correlation analyses performed in a small cohort, the risk of type I error is increased; therefore, findings should be interpreted cautiously and considered exploratory.

No formal correction for multiple comparisons was applied due to the exploratory nature of this pilot study; therefore, *p*-values should be interpreted descriptively.

### 4.6. Study Limitations

This study has several inherent limitations. First, the sample size was small (n = 20), which may affect statistical power and limit the generalizability of findings. Second, the single-center design and specific population may limit external validity. Third, dynamic hormonal testing (e.g., insulin tolerance test, ACTH stimulation test) could not be performed due to the hemodynamic instability and altered consciousness of patients during the acute phase, potentially leading to underdiagnosis of subtle hormonal deficiencies. Fourth, long-term follow-up data were not available to assess the persistence or resolution of hormonal abnormalities. Finally, the study lacked a control group of patients without SAH, limiting causal inferences.

## 5. Conclusions

This prospective pilot study suggests that greater clinical severity in aneurysmal subarachnoid hemorrhage is associated with a higher frequency of acute biochemical hypothalamic–pituitary axis abnormalities, particularly involving the ACTH axis and multiple hormonal pathways. Among the hormonal axes assessed, ACTH-axis abnormalities showed the strongest association with neurological severity scores.

However, given the small sample size, reliance on basal hormone measurements, absence of dynamic endocrine testing, and lack of longitudinal follow-up, these findings should be interpreted as exploratory and hypothesis-generating rather than diagnostic of true pituitary insufficiency.

Larger multicenter prospective studies incorporating dynamic hormonal testing and long-term endocrine follow-up are required to determine whether these early biochemical abnormalities represent transient stress responses or clinically meaningful pituitary abnormalities after aSAH.

## Figures and Tables

**Table 1:** Prevalence and mean levels of hormonal (biochemical) abnormality.

Hormone	Mean Level (±SD)	Reference Range (Institutional/Healthy Adult)	Biochemical Abnormality Prevalence
GH	0.79 ± 0.41 ng/mL	0.5–2.47 ng/mL	5% (1/20)
TSH	0.91 ± 0.62 μIU/mL	0.27–4.2 μIU/mL	40% (8/20)
ACTH	13.9 ± 18.7 pg/mL	7.2–63.3 pg/mL	75% (15/20)
ADH (FIA)	68.2 ± 42.1 pg/mL	0.87–30.58 pg/mL *	50% (10/20)
ADH (FPD)	295.6 ± 8.3 mOsmol/kg	280–300 mOsmol/kg	0% (0/20)

* Note: Unit conversion may be required for direct comparison. Conversion factor: 1 pg/mL ≈ 0.926 pmo/L. Therefore, 68.2 pg/mL ≈ 63.2 pmol/, which is above the upper reference limit, indicating relative ADH elevation. However, concurrent plasma osmolality (FPD) was normal in all patients, suggesting appropriate ADH response rather than true vasopressin excess or deficiency. No critical illness-adjusted ranges were used.

**Table 2:** Spearman correlation coefficients between hormonal abnormalities and severity metrics.

Hormone Deficiency	GCS	Hunt-Hess Scale	Modified Fisher Scale
ACTH	ρ = −0.61 ** (*p* = 0.004)	ρ = 0.59 ** (*p* = 0.006)	ρ = 0.41 (*p* = 0.065)
TSH	ρ = −0.31 (*p* = 0.12)	ρ = 0.29 (*p* = 0.23)	ρ = 0.22 (*p* = 0.17)
GH	ρ = −0.42 * (*p* = 0.048)	ρ = 0.39 * (*p* = 0.033)	ρ = 0.33 (*p* = 0.058)
ADH	ρ = −0.38 (*p* = 0.073)	ρ = 0.34 * (*p* = 0.045)	ρ = 0.28 (*p* = 0.084)
Multiple Axes	ρ = −0.68 ** (*p* = 0.001)	ρ = 0.72 ** (*p* < 0.001)	ρ = 0.57 ** (*p* = 0.009)

* *p* < 0.05; ** *p* < 0.01.

**Table 3:** Comparison of severity scores by hormonal abnormality status.

Deficiency Group	GCS (Mean ± SD)	Hunt-Hess (Mean ± SD)	Fisher (Mean ± SD)
No Deficiency (n = 3)	13.7 ± 1.5	2.0 ± 0.8	2.3 ± 0.6
Single Axis (n = 7)	11.4 ± 2.3	2.9 ± 1.1	2.7 ± 0.9
Multiple Axes (n = 10)	8.2 ± 2.8 **	4.1 ± 0.9 **	3.4 ± 0.7 *

* *p* < 0.05; ** *p* < 0.01 compared to no abnormality group.

## Data Availability

The data presented in this study are available on request from the corresponding author. The data is not publicly available due to privacy and ethical restrictions.

## References

[1] Etminan N., Chang H.S., Hackenberg K., de Rooij N.K., Vergouwen M.D.I., Rinkel G.J.E., Algra A. (2019). Worldwide Incidence of Aneurysmal Subarachnoid Hemorrhage According to Region, Time Period, Blood Pressure, and Smoking Prevalence in the Population: A Systematic Review and Meta-analysis. JAMA Neurol..

[2] Claassen J., Park S. (2022). Spontaneous subarachnoid haemorrhage. Lancet.

[3] Etminan N., Macdonald R.L. (2017). Management of aneurysmal subarachnoid hemorrhage. Handb. Clin. Neurol..

[4] Rautalin I., Volovici V., Stark B.A., Johnson C.O., Kaprio J., Korja M., Krishnamurthi R.V., Nair B.S., Ranta A., GBD 2021 Global Subarachnoid Hemorrhage Risk Factors Collaborators (2025). Global, Regional, and National Burden of Nontraumatic Subarachnoid Hemorrhage: The Global Burden of Disease Study 2021. JAMA Neurol..

[5] Hannon M.J., Crowley R.K., Behan L.A., O’Sullivan E.P., O’Brien M.M., Sherlock M., Rawluk D., O’Dwyer R., Tormey W., Thompson C.J. (2013). Acute glucocorticoid deficiency and diabetes insipidus are common after acute traumatic brain injury and predict mortality. J. Clin. Endocrinol. Metab..

[6] Khajeh L., Blijdorp K., Heijenbrok-Kal M.H., Sneekes E.M., van den Berg-Emons H.J., van der Lely A.J., Dippel D.W., Neggers S.J., Ribbers G.M., van Kooten F. (2015). Pituitary dysfunction after aneurysmal subarachnoid haemorrhage: Course and clinical predictors—The HIPS study. J. Neurol. Neurosurg. Psychiatry.

[7] Ntali G., Tsagarakis S. (2020). Pituitary dysfunction after traumatic brain injury: Prevalence and screening strategies. Expert Rev. Endocrinol. Metab..

[8] Hijdra A., Van Gijn J., Stefanko S., Van Dongen K.J., Vermeulen M., Van Crevel H. (1986). Delayed cerebral ischemia after aneurysmal subarachnoid hemorrhage: Clinicoanatomic correlations. Neurology.

[9] Klose M., Brennum J., Poulsgaard L., Kosteljanetz M., Wagner A., Feldt-Rasmussen U. (2010). Hypopituitarism is uncommon after aneurysmal subarachnoid haemorrhage. Clin. Endocrinol..

[10] Pluta R.M., Hansen-Schwartz J., Dreier J., Vajkoczy P., Macdonald R.L., Nishizawa S., Kasuya H., Wellman G., Keller E., Zauner A. (2009). Cerebral vasospasm following subarachnoid hemorrhage: Time for a new world of thought. Neurol. Res..

[11] Kelly D.F., Gonzalo I.T., Cohan P., Berman N., Swerdloff R., Wang C. (2000). Hypopituitarism following traumatic brain injury and aneurysmal subarachnoid hemorrhage: A preliminary report. J. Neurosurg..

[12] Schneider H.J., Kreitschmann-Andermahr I., Ghigo E., Stalla G.K., Agha A. (2007). Hypothalamopituitary dysfunction following traumatic brain injury and aneurysmal subarachnoid hemorrhage: A systematic review. JAMA.

